# Combined lithotripsy of mechanical clamping and electrohydraulics in facilitating endoscopic management of refractory residual biliary calculi after surgery

**DOI:** 10.1038/s41598-020-58394-9

**Published:** 2020-02-13

**Authors:** Xu-dong Wen, Li-na Ren, Tao Wang, Xiao-juan Wang, Nalu Navarro-Alvarez, Liang-ping Li, Wei-hui Liu

**Affiliations:** 1 Department of Gastroenterology, Chengdu First People’s Hospital, Chengdu, Sichuan Province 610016 China; 2 Department of Gastroenterology and Hepatology, Sichuan Academy of Medical Sciences & Sichuan Provincial People’s Hospital, Chengdu, Sichuan Province 610072 China; 3 The General Hospital of Western Theater Command, Chengdu, Sichuan Province 610083 China; 4 Department of Surgery, Massachusetts General Hospital, Harvard Medical School, Boston, Massachusetts 02138 USA

**Keywords:** Choledocholithiasis, Bile ducts

## Abstract

Although postoperative cholangioscopy (POC) guided electrohydraulic lithotripsy (EHL) is considered to be a conventional technique for residual biliary calculi, its efficacy still needs to be improved to fit in the managemet of refractory calculi. This study evaluated the efficacy and safety of combined lithotripsy of mechanical clamping and electrohydraulics in fragmentation and removal of refractory calculi. Totally, 281 patients, who suffered from residual biliary calculi after hepatectomy and underwnet POC from August 2016 to June 2018 were involved. The first 128 patients were subjected to conventional EHL, and later consective 153 to combined lithotripsyof mechanical clamping and EHL. Perioperative data, technical information, treatment outcomes and follow-up results were collected. Clinical characteristics were statistically comparable (P > 0.05). The overall POC interventional sessions (2.0 ± 0.65 vs. 2.9 ± 1.21 sessions), average operating time (99.1 ± 34.88 vs. 128.6 ± 72.87 minutes), incidence of intraoperative hemobilia (4.58% vs. 10.93%), cholangitis (6.54% vs. 14.06%), postoperative complications (10.45% vs. 21.87%), T-tube retaining time after first POC (20.7 ± 5.35 vs. 28.1 ± 8.28 days), and treatment costs ($2375 ± 661.72 vs. $3456.7 ± 638.07) were significantly lower in the combined lithotripsy group than those in the EHL group (P < 0.05). There were no differences between the two groups in calculi recurrence at half-a year, or one year follow-up. In conclusion, combined lithotripsy of mechanical clamping and electrohydraulics can safely and effectively benefit postoperative patients along with refractory residual biliary calculi.

## Introduction

Although biliary calculus is a benign disease characterized for the presence of intrahepatic bile duct calculi, it may cause serious complications^1,2^. Hepatectomy combined with intraoperative cholangioscopy is regarded as the most helpful approach not only for the removal of the intrahepatic calculi and lesions, but also for reducing the risk of calculi recurrence and cholangitis^3^. Unfortunately, this technique is not suitable for complicated calculi distributing bilobarly or diffusely^4^. In management of complicated calculi, placement of a T-tube drainage for further postoperative cholangioscopy (POC) was feasible, which allows for repeated sessions for calculi extraction^5,6^.

When dealing with complicated residual biliary calculi such as with large size, hard consistency, or anatomical abnormalities, conventional POC managements including perfusion and basket extraction are often difficult, or even impossible in patients with impacted calculi^7^. Among advanced therapeutic strategies including electrohydraulic lithotripsy (EHL), laser lithotripsy, pneumatic lithotripsy and hyperacoustic lithotripsy, the EHL being the most commonly used in clinic because of its efficiency and low costs^8–11^. It allows calculi to be crushed by energy absorption, which leads to a build-up of pressure gradients and the formation of shear and tear forces^9^.

**Figure 1:**
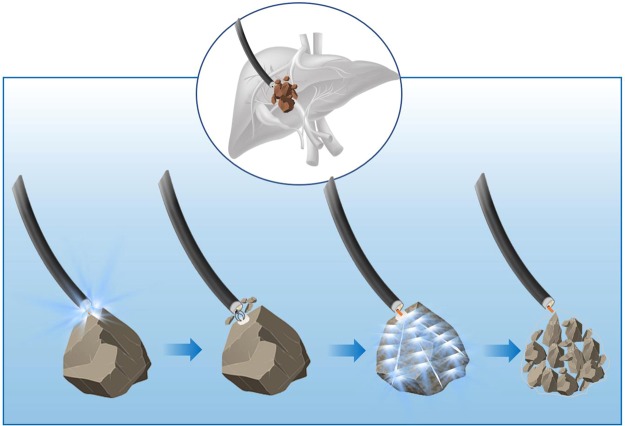
The schematic diagram of combined lithotripsy of mechanical clamping and electrohydraulics.

Though POC guided EHL is generally used as an active measure for the treatment of residual biliary calculi, its efficiency needs to be promoted especially in dealing with refractory calculi^12^. It is embarrassed that calculi with hard property impacted in the intrahepatic bile ducts, at the duodenal papilla, or in the extraction basket are unable to be fragmented despite repeated and long-term exposure to EHL. What’s more, the diffused energy from long-term EHL may cause persistent damage to the wall of bile duct, which in turn results in serious complications including perforation and bleeding. Therefore, an effective and practical alternative procedure is urgently required. To solve this problem, we innovatively combined mechanical clamping with EHL, in where biopsy forceps were introduced to pre-break the hard surface of calculus (Fig. 1). By clamping, it generated a pothole on the surface to both prevent the shock wave energy of EHL from scattering and focus it within the calculus, which eventually caused fragmentation. This retrospective study was designed to investigate the efficiency and security of this combined lithotripsy (CL) in treating refractory calculi in patients with residual biliary calculi.

## Results

### Demographic and clinical characteristics

Gender, age, biochemical parameters, Child-Pugh classification, and total surgical modalities were similar between the CL and EHL groups (*P* > 0.05) (Table 1). In biochemical parameters, index of platelet, alanine aminotransferase, aspartate transaminase and total bilirubin in two groups were almost normal and comparable. While, the index of C reaction protein was relatively high (18.5 ± 13.47 mg/L in the CL group, and 17.2 ± 14.56 mg/L in the EHL group. Most patients in the two groups were classified as Child-Pugh A. From the 148 patients that underwent choledocholithotomy and T-tube drainage (CLT), 83 were in the CL and 65 in the EHL group; whereas from the 133 patients that underwent choledochojejunostomy and T-tube drainage (CJT), 70 were in the CL and 63 in the EHL group.

**Table 1:** Baseline characteristics of patients.

Variable	CL group (n = 153)	EHL group (n = 128)	*P*
**Sex, No. (%)**			0.734
Male	71 (46.41)	62 (48.44)	
Female	82 (53.59)	66 (51.56)	
**Age (yr)**	51.5 ± 11.55	51.9 ± 12.41	0.804
**Biochemical parameters**			
Platelet ( × 10^9^/L)	185.0 ± 53.33	193.0 ± 62.64	0.255
CRP (mg/L)	18.5 ± 13.47	17.2 ± 14.56	0.441
ALT (U/L)	39.3 ± 15.33	41.0 ± 16.21	0.370
AST (U/L)	32.5 ± 17.45	31.7 ± 19.68	0.721
Albumin (g/L)	39.3 ± 8.87	40.2 ± 9.37	0.412
TBIL (μmol/L)	12.5 ± 7.03	13.7 ± 11.57	0.306
**Child-Pugh classification, No. (%)**			0.097
A	133 (86.93)	119 (92.97)	
B	20 (13.07)	9 (7.03)	
**Surgical modalities, No. (%)**			0.562
Hepatectomy plus CLT	83 (54.25)	65 (50.78)	
Hepatectomy plus CJT	70 (45.75)	63 (49.22)	

Abbreviations: CL = combined lithotripsy of mechanical clamping and electrohydraulics; EHL = electrohydraulic lithotripsy; CLT = choledocholithotomy and T-tube drainage; CJT = choledochojejunostomy and T-tube drainage; CRP = C reactive protein; ALT = alanine aminotransferase; AST = aspartate transaminase; TBIL = total bilirubin.

### Residual calculi characteristics

The calculi distribution between the CL group and EHL group was statistically comparable with no significant difference in the number or size of calculi (*P* > 0.05) (Table 2). The most common type of calculi in both groups was brown pigment (52.94% in CL group, and 49.22% in EHL group). Black pigment, and cholesterol calculi were 35.94% and 11.11% respectively in the CL group, and 35.16%, and 15.63% in the EHL group. Impactions in hepatic bile duct (IHD), common bile duct (CBD), or in basket during the procedure were also recorded and they were all similarly observed.

**Table 2:** Residual calculi condition between two groups.

Variable	CL group	EHL group	*P*
**Calculi distribution, No**.			0.977
Right anterior lobe (V + VIII)	56	41	
Right posterior lobe (VI + VII)	50	40	
Left internal lobe (IV)	47	42	
Left external lobe (II + III)	64	52	
Caudate lobe (I)	21	19	
Common bile duct	23	16	
**Calculi Numbers**^**#**^	3.1 ± 1.42	3.2 ± 1.43	0.346
**Calculi Property, No. (%)**			0.528
Brown pigment	81 (52.94)	63 (49.22)	
Black pigment	55 (35.94)	45 (35.16)	
Cholesterol	17 (11.11)	20 (15.63)	
**Maximum diameter (mm)**	31.8 ± 7.78	32.8 ± 8.92	0.298
**Calculi Impaction, No**.			0.706
Intrahepatic duct	105	88	
Common bile duct	16	11	
Basket	22	14	

Abbreviations: CL = combined lithotripsy of mechanical clamping and electrohydraulics; EHL = electrohydraulic lithotripsy. Couinaud classification of hepatic segments is shown in Roman numerals.

### Intraoperative and postoperative results

All calculi were almost extracted after several POC interventions with lithotripsy, and no death occurred during or after POC in neither of the two groups. In contrast, overall POC sessions and operating time using lithotripsy techniques were significantly fewer and shorter in the CL than in the EHL group (*P* = 0.000) (Table 3). In addition, the required saline volume in the CL group was less than in the EHL group (*P* = 0.000).

**Table 3:** Intraoperative results between the two groups.

Variable	CL group	EHL group	*P*
Overall POC sessions^#^	2.0 ± 0.65	2.9 ± 1.21	0.000^‡^
Overall operating time (min)^#^	99.1 ± 34.88	128.6 ± 72.87	0.000^‡^
Injected physiological saline volume (mL)^#^	4544.4 ± 1149.10	6472.2 ± 2426.52	0.000^‡^
Hemobilia, No. (%)	7 (4.58)	14 (10.93)	0.043^*^
Cholangitis, No. (%)	10 (6.54)	18 (14.06)	0.036^*^
Clearance rate (%)	98.70	98.44	1.000

Abbreviations: CL = combined lithotripsy of mechanical clamping and electrohydraulics; EHL = electrohydraulic lithotripsy. ^#^Per patient. ^*^P < 0.05, ^‡^*P* < 0.001.

Hemobilia (*P* = 0.043) and cholangitis (*P* = 0.036) were observed to a lessen extend in the CL group than those in the EHL group. Among the patients with hemobilia, 90.00% (9/10) stanched by hemostasis in the CL group, and 94.44% (17/18) in the EHL group. The overall postoperative complication rates were significantly lower in the CL than EHL group (*P* = 0.009) (Table 4). Incidence rates of cholangitis (*P* = 0.036), diarrhea (*P* = 0.041), and hemobilia (*P* = 0.036) were significantly lower in the CL when compared with the EHL group (Table 4). No significant difference was found in bile leakage, jaundice, postoperative bleeding, wound infection, and sinus perforation between two groups. Detailly, the distribution of complications in different impacted locations was analyzed. In the EHL group, intraoperative complication rates significantly differed among the different locations (*P* = 0.011), and complication rate in basket impaction was obliviously higher than those in IHD and CBD (Supplementary table S1). Though there existed no significant differences in postoperative complication among different locations in the EHL group, the occurrences of total postoperative complication (42.86%) and postoperative cholangitis (35.71%) were relatively high. The complication rates among different locations in the CL group were comparable (*P* > 0.05). Comparing with the EHL group, the CL group had significantly lower total postoperative rate (*P* = 0.029) and occurrence of postoperative diarrhea (*P* = 0.044) in the IHD impaction cases (Supplementary table S2), and less total intraoperative complication (*P* = 0.005), intraoperative cholangitis (P = 0.036), and postoperative cholangitis (*P* = 0.024) in the Basket impaction cases.

**Table 4:** Postoperative complications between the two groups.

Variable	CL group	EHL group	*P*
**Overall complication, No. (%)**	16 (10.45)	28 (21.87)	0.009^†^
**Presentation, No. (%)**			
Acute cholangitis	10 (6.54)	18 (14.06)	0.036^*^
Diarrhea	12 (7.84)	20 (15.63)	0.041^*^
Hemobilia	3 (1.96)	9 (7.03)	0.036^*^
Postoperative bleeding	3 (1.96)	3 (2.34)	0.985
Bile leakage	3 (1.96)	5 (3.91)	0.537
Jaundice	5 (3.27)	7 (5.47)	0.854
Wound infection	4 (2.61)	5 (3.91)	0.723
Sinus perforation	2 (1.31)	1 (0.78)	0.985

Abbreviations: CL = combined lithotripsy of mechanical clamping and electrohydraulics; EHL = electrohydraulic lithotripsy. ^*^P < 0.05, ^†^*P* < 0.01.

### Costs and follow-up results

After the first time of POC, the T-tube retaining time significantly differed between the two groups (20.7 ± 5.35 days in the CL *vs*. 28.1 ± 8.28 days in the EHL; *P* = 0.000) (Table 5). Of the two groups, 8 patients with bleeding (4 in CL and 4 in EHL) and 8 with bile leakage (3 in CL and 5 in EHL) were hospitalized. Among the hospitalized patients, one in each group underwent exploratory laparotomy, and 7 received transcatheter arterial embolization therapy. Cost was significantly lower in the CL group than that in the EHL group (*P* = 0.000) (Table 5).

**Table 5:** Treatment information between the two groups.

Variable	CL group	EHL group	*P*
T-tube retaining time after first POC (day)	20.7 ± 5.35	28.1 ± 8.28	0.000^‡^
Hospitalization conversion, No. (%)	7 (3.27)	9 (6.25)	0.236
Surgery conversion, No. (%)	1 (0.65)	1 (0.78)	1.000
TAE conversion, No. (%)	3 (1.96)	4 (3.13)	0.811
Treatment costs (USD)	2375.6 ± 661.72	3456.7 ± 638.07	0.000^‡^

Abbreviations: CL = combined lithotripsy of mechanical clamping and electrohydraulics; EHL = electrohydraulic lithotripsy; TAE = transcatheter arterial embolization. ^‡^*P* < 0.001.

The follow-up time ranged from 2 to 12 months. Median follow-up was 10.7 ± 2.67 months in the CL and 10.34 ± 2.33 months in the EHL group (*P* = 0.215). For biliary calculi, recurrence at half-year was statistically similar (5.23% in the CL group *vs*. 5.46% in the EHL group; P = 0.929), as well as the one-year recurrence (9.15% in the CL group *vs*. 10.94% in the EHL group; P = 0.618).

### Procedure-related serious complications

**Figure 2:**
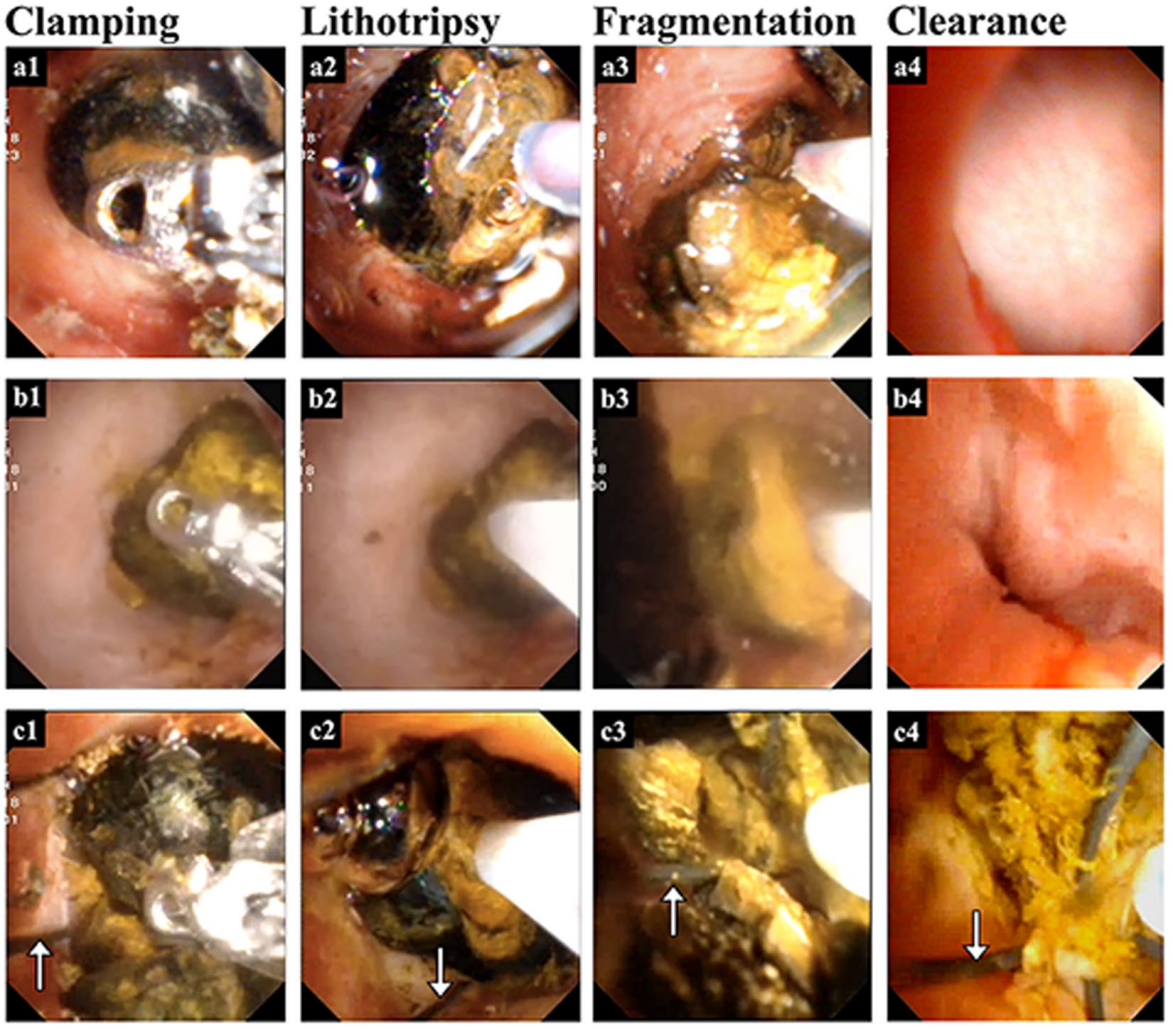
Three specific applications of combined lithotripsy (CL) of mechanical clamping and electrohydraulics in management of the refractory biliary calculi. (**a**) CL was applied when calculus was impacted within the intrahepatic bile duct. (**b**) CL facilitated calculus extraction while calculus got stuck in Sphincter of Oddi. (**c**) CL helped withdraw the impacted basket when it held big calculus (arrow indicated the basket wire).

Bile ductal perforation and mucosal damage, causing hemorrhage and bile leakage, as well as sphincter of oddi dysfunction when calculi are impacted in the duodenal papilla, or even cholangitis or pancreatitis are the most frequent complications of EHL^13–15^. The use of CL properly enables refractory calculi fragmentation to prevent these serious complications. In this study, CL was used in difficult cases when calculi were impacted in the intrahepatic ducts (Fig. 2a), in the duodenal papilla (Fig. 2b) or in the extraction basket (Fig. 2c).

## Discussion

EHL is commonly used in clinic, and its generated high-amplitude frequency hydraulic pressure waves could produce high energy that is absorbed by stones aiding in the disintegration of calculus^16,17^. In management of refractory biliary calculi, conventional EHL has difficulties in dealing with them efficiently, due to their peculiar consistency, size, location, and so on^18^. The low efficiency of EHL, on the one hand, leads to increased POC duration, and extra POC interventions; on the other hand, results in prolonged T-tube retaining time, and accessorial treatment costs^19^. In addition, it is reported that the low calculi removal efficacy resulted in severe liver damage, apart from more patients suffering and higher costs^20^. Therefore, there is an increasing need to improve the validity and safety of successful EHL therapy.

After the observation of rock blasting process in quarry, we found that the bomb was buried inside of the hard rock by drilling a tunnel to avoid the energy scattering. For the same reason, in the EHL process, whether a hole could be made to concentrate the energy? In clinic, biopsy forceps clamping has been routinely used for lesion biopsy or tiny calculi extraction. Based on biopsy forceps’ characteristic, we creatively used the mechanical clamping to pre-break the tough calculi’s surface as to facilitate further EHL. The created pothole helped not only to reduce the energy dispersion, but also to strengthen the energy absorption within the calculi.

Due to the biopsy forceps’ mechanical clamping, CL strategy greatly improved the outcome of EHL in managing refractory residual calculi. Though the total clearance rates were comparable, the fewer intra/post-operative complications were significantly observed in CL group than in conventional EHL group, as well as less suffering and costs for patients. In the operative process, 4.58% hemobilia and 6.54% cholangitis occurred in CL group, less than half of those (10.93%, and 14.06%) in EHL group. The significant differences might result from the decreased POC sessions and shortened operating time. Wong *et al*. reported that 12% of cases developed cholangitis by laser lithotripsy of complicated biliary stones^21^. Moreover, potential risks for postoperative complication were reduced in CL group (10.45%), in terms of its relatively high efficiency and the shortened T-tube retaining time accordingly. A similar report indicated that complications occurred more than 22.2% in all stages^22^. It was also reported complication rate of 19% and mortality of 3% in endoscopic removal of retained stones after biliary surgery^23^. The simultaneous descent in postoperative cholangitis and hemobilia by CL strategy also proved its high efficiency in calculi lithotripsy when compared with EHL^24^. While in conventional EHL way, spark with higher voltage might result in post-procedure complications including cholangitis, hemobilia, and bile leak^25^. For example, when calculi with hard consistency impacted in the basket, repeated EHL with high voltage would cause serious risk of thermal ductal wall damage, since the heated metal wire might dissipate heat, or directly served as an energy conductor to the ductal mucosa. This predicament could be well avoided by the CL method. In addition, the analysis of the relation between complications and impacted calculi distributions indicated that, on the one hand, basket impaction mainly contributed to the increased complication in EHL group; on the other hand, the high efficacy of CL led to reduced complications mainly in IHD and basket impactions when compared with EHL technique. As for complications occurred in the study were all promptly managed. Most intraoperative hemobilia could be managed by irrigation of cold physiological saline solution or epinephrine. Only one in each group was converted to hospitalization for intraoperative hemobilia reason. While intraoperative cholangitis was observed, the operation should be immediately stopped and replacement of T-tube was performed for the next session 14 days later. Cholangitis related symptoms could generally be improved after 2 hours’ rest. The incidence of postoperative diarrhea was lower in the CL group, since the reduced amount irrigation of physiological saline could be a protective factor^26^. For patients who were dehydrated, intravenous fluid therapy was performed. Patients with delayed bleeding and bile leakage were all converted to hospitalization. Empirically, drugs, arterial embolization, and laparotomy were gradually used for delayed bleeding; while, bile leakage was generally managed with percutaneous drainage directed by ultrasound. Wound infection might extend the time of T-tube placement, and sinus perforation gradually healed after the T-tube removal.

The CL is efficient, and it is indeed one-step forward on the base of EHL. Firstly, the CL could be easily performed under direct visual control using various endoscopic apparatus, such as POC, percutaneous transhepatic cholangioscopy, peroral cholangioscopy, and SpyGlass Direct Visualization System^5,27–29^. In case conventional EHL was unsuccessful in the treatment of refractory calculi, CL could be further commonly used. Secondly, the removal of the calculi’s surface could be easily done with biopsy forceps through the accessory channel. A direct visualization makes mechanical clamping a safer procedure with minimal mucosa and duct injury^22^. Since clamping by biopsy forceps was a routine process in lesion biopsies, such as bile duct^29,30,31^, lung^32^, esophagus^33^, and no additional training is needed, therefore endoscopists could easily master the CL technique. Finally, the low cost of the biopsy forceps made CL an attractive alternative to minimize the procedure costs for the patients. In the study, the cost analysis indicated that the reduced overall POC sessions and complication rates contributed to the medical cost cutting for patients. Actually, various measures of lithotripsy might be considered when EHL fails, and laser lithotripsy was a well-established method commonly used in multiple centers^34,35,36^. However, laser lithotripsy was reported with limitations of higher costs due to an expensive equipment, poor exploding force, and fragility of the probe^36,37^. Effective and affordable medical approaches are needed to maximally benefit the patients. Considering the above situations, CL was mainly considered in our center, for that the cost of biopsy forceps used in our CL was much less than that of laser lithotripsy.

Though we have proven that CL provided superiorities in the intraoperative and postoperative results compared with EHL, the present study had some limitations. First, the efficiency of CL was not compared with other measures of lithotripsy. In addition, the best timing for introducing the biopsy forceps into the EHL to perform the clamping remains to be solved. Although clinical baseline data from the two groups was comparable, patients were not randomly selected as a result of the retrospective nature of this study. Based on these limitations, studies with larger sample size, prospective design, and multicenter randomized controlled trials are needed.

## Methods

### Patients

This study was performed in accordance with clinical study protocols and the principles of the Declaration of Helsinki (modified 2000) and approved by the Research Care and Ethics Committee of our institution (No. SPPHCT2018–MED-11). Patients or their families gave signed informed consent participate in this study. All patients with residual biliary calculi after hepatectomy presenting to the Endoscopic Department from August 2016 to June 2018 were included in this retrospective study. These patients either underwent CJT or CLT. A total of 281 consecutive patients who received cholangioscopy-guided EHL in POC treatment were included. The first 128 patients involved from August 2016 to June 2017 underwent traditional EHL. Whereas the later 153 patients obtained from June 2017 to June 2018 were all subjected to CL, as we gradually observed the usefulness of clamping in assisting EHL in the treatment of difficult stones.

The inclusion criteria used for this study were the following: (1) patients with a first episode of hepatolithiasis that underwent hepatectomy with a T-tube drainage; (2) patients with a diagnosis of residual biliary calculi by imaging and cholangioscopy; (3) patients with characteristics of refractory calculi (a. impacted calculus; b. not impacted but the diameter of calculus was > 15 mm and had hard consistency); (4) patients requiring EHL in POC treatment. Exclusion criteria were listed as below: (1) less than 4 weeks of T-tube indwelling for sinus maturation; (2) general conditions contraindicating the POC, or signs of uncontrolled cholangitis (fever, shivering, abdominal pain, progressive jaundice); (3) residual calculi in grade III or above bile branches which cholangioscopy is basically not feasible; (4) tumor invading the bile duct, duodenal papilla, or anastomotic stoma; (5) age under 18 years or over 80 years.

### Procedures

#### Diagnosis and procedure preparation

Following identification of the residual biliary calculi intraoperatively, imaging methods, such as ultrasound, computed tomography (CT), or T-tube radiography were used for the diagnosis and confirmation of the calculi location, which was assessed by two or more experienced radiologists. All patients were subjected to routine blood test including coagulation factors, C reactive protein, and liver function index before POC. Fasting of 4 to 8 hours was required. Before surgery, all patients were given pethidine (50 mg) by intramuscular injection and positioned supine.

#### Postoperative Cholangioscopy (POC)

All procedures were performed in our center by experienced endoscopists. The diameter of the electronic cholangioscopy used in this study was 4.8 mm (CHF-V, Olympus, Japan). Before dealing with calculi, if strictures in bile ducts were found, dilatation and stenting was first considered. Silt like calculi were cleared by irrigation and suction, whereas small sized calculi were removed by extraction baskets (JHY-BAS-18-70-15-N4-B-O, Jiuhong, China), or grasping forceps (JHY-FG-23-160-A4, Jiuhong, China). Large stones needed fragmentation by electrohydraulic lithotripter (DLZ-1, Yidalong, China) prior to removal in order to prevent impaction. Stone fragments were extracted through basket transportation or by simultaneous irrigation and suction. When possible, small calculi were pushed into enteric cavity through the duodenal papilla or anastomotic stoma. In the study, the manipulation time per session was strictly set as 2 hours to avoid the sharply increased complications, which was generally regulated in clinical work. Ultrasound, T-tube radiography and cholangioscopy were routinely used to confirm the absence of calculi before removal of the T-tube drainage.

#### Combined lithotripsy of mechanical clamping and electrohydraulics

**Figure 3:**
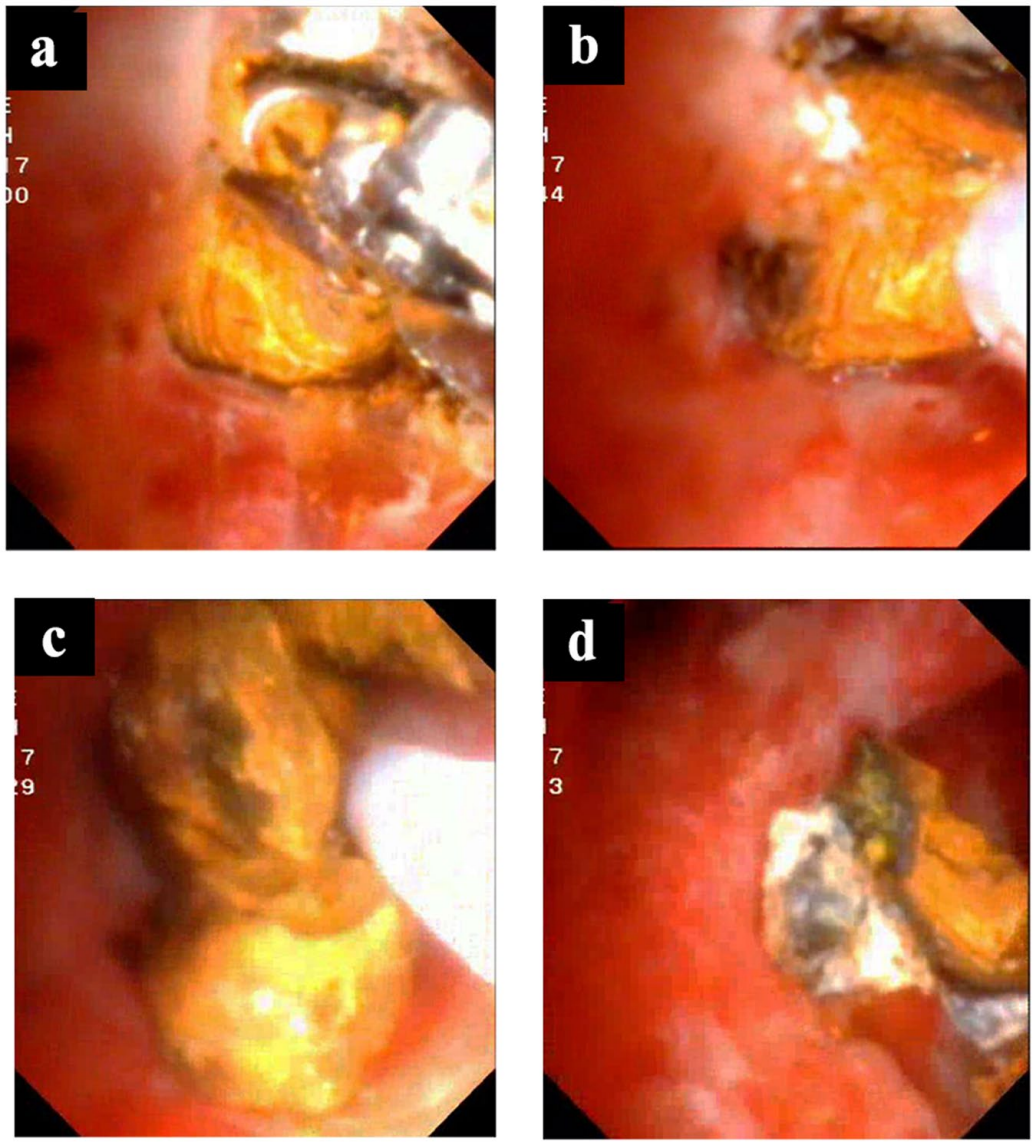
Application of combined lithotripsy of mechanical clamping and electrohydraulics in refractory calculus. (**a**) Impacted calculus with hard texture failed to be fragmented by direct electrohydraulic lithotripsy (EHL). (**b**) Biopsy forceps pre-broke the surface of the calculus by clamping. (**c**) The EHL probe accurately targeted the exposed area of calculus. (**d**) The calculus was easily fragmented into pieces by short-time EHL. (**e**) Impacted calculus was totally cleared.

EHL system was constituted by a bipolar probe, which was connected to a charge generator. The probe of the EHL fiber is inserted into the accessory channel of a cholangioscopy until protruded about 2–3 mm from the scope. The voltage was set to 70–90 V and 10 shocks per second were used. Sparks produced high-amplitude hydraulic pressure waves for stone fragmentation. The probe was positioned enface with the calculus, while the generator’s foot pedal was depressed to deliver energy. When one refractory calculus could not be fragmented by 2 minutes of continuing EHL, a pair of biopsy forceps was introduced to first break the surface of the calculus (Fig. 3a). The probe was then placed against the weak area exposed by clamping (Fig. 3b), and further EHL was performed to successfully fragment the calculus (Fig. 3c). Finally, the fragmented calculi were completely extracted by the basket (Fig. 3d). The removal of the impacted calculus was considered complete, when exiguous fragments were cleared.

### Clinical and treatment parameters

All patients underwent ultrasound, CT, and routine blood tests. Distribution of residual calculi was recorded, as well as the maximum diameter of calculi. The calculi’s properties were classified on the basis of visual appearance. Brown pigment calculi mainly containing calcium bilirubinate, had a rough and hard surface and presented an interphase of yellow and white within it. Black pigment calculi had a smooth, rough, and black-colored surface, surrounding a yellow cholesterol-rich inner body. Cholesterol calculi displayed a gray-white appearance. The calculi properties were all finally authenticated by pathology. Calculi diameter was estimated by comparing the diameter of the stone with that of the tip of the endoscope, as measured on the imaging results. In addition, the number and condition of impactions in each session were also recorded. During POC, intraoperative results were analyzed, including number of sessions, operating time, and patients’ presentation. The treatment costs during hospitalization were also calculated, including medical examination, device, accessories, procedure, hospitalization, and medications. Blood biochemical tests were taken upon admission and were followed up every 3 months during the first year after T-tube sealing, and every 4 months during the second year. Residual calculi were defined as the presence of calculi in the biliary duct within 3 months after T-tube removal; recurrent stones or episodes of cholangitis occurring 3 months later after sinus closure were defined as recurrence.

### Definitions of complications

Occurrence of complications was collected for the calculation of complication rates. Different presentations of complications occurring in one patient were recorded as one, and the same presentation occurred in different sessions was also defined as one. Intraoperative hemobilia was defined as the observation of mucosal bleeding under direct cholangioscopic vision, and post-POC hemobilia was defined as the presence of non-congealable drainage from the T-tube. Post-POC delayed bleeding was defined as clinical evidence of bleeding, such as melena, hematemesis or hematochezia, or a decrease in hemoglobin by more than 2 g/dL from the baseline within 14 days of operation^38^. Hemobilia several hours after the lithotripsy indicated the delayed bleeding. Patients who suffered from epigastric pain, fever, and tremble were considered to have cholangitis. Diarrhea is defined as the condition of having at least three loose, liquid, or watery bowel movements after the POC^39,40^. Bile leakage was defined as high total bilirubin or amylase levels in the abdominal drains (> 3 times serum levels). Wound infection was defined as the presence of bacteria from the wound. Sinus perforation was defined as the presence of links between sinus and enterocoelia or bile ducts.

### Statistical analyses

All statistical analyses were performed using SPSS21.0 (IBM SPSS, USA). All data were presented as percentage of appropriate groups or mean ± standard deviation. Statistical comparisons were done with t tests or Wilcoxon rank sum tests. Rates were compared using Chi-square test, continuity correction test, or Fisher exact test.

## Supplementary information

Supplementary information.
