# Supplementary material for: Combined lithotripsy of mechanical clamping and electrohydraulics in facilitating endoscopic management of refractory residual biliary calculi after surgery
Source: Sci Rep. 2020 Feb 13;10:2604. doi: 10.1038/s41598-020-58394-9 (PMC7018823; doi:10.1038/s41598-020-58394-9)
Supplement: Supplementary file 1 — Supplementary information. [file 41598_2020_58394_MOESM1_ESM.pdf]

Combined lithotripsy of mechanical clamping and electrohydraulics in facilitating endoscopic management of refractory residual biliary calculi after surgery.

Xu-dong Wen MD., Li-na Ren MD., Tao Wang MD, Ph.D., Xiao-juan Wang MD., Nalu Navarro-Alvarez MD, Ph.D., Liang-ping Li MD., Wei-hui Liu MD, Ph.D.

Supplementary information

Supplementary table S1. Complications among the different impacted positions in two groups.

**Variable**	**EHL group**			***P***	**CL group**			***P***
	**IHD (n=88)**	**CBD (n=11)**	**Basket (n=14)**		**IHD (n=105)**	**CBD (n=16)**	**Basket (n=22)**	
**Intraoperation, No. (%)**	18 (20.45)	2 (18.18)	8 (57.14)	0.011*	11 (10.48)	1 (6.25)	2 (9.09)	0.863
Hemobilia	10 (11.36)	2 (18.18)	2 (14.29)	0.790	5 (4.76)	1 (6.25)	1 (4.54)	0.964
Cholangitis	11 (12.50)	0	6 (42.86)	0.004†	7 (6.67)	0	2 (9.10)	0.499
**Postoperation, No. (%)**	17 (19.31)	4 (36.36)	6 (42.86)	0.094	9 (8.57)	2 (12.5)	3 (13.64)	0.712
Cholangitis	12 (13.63)	1 (9.09)	5 (35.71)	0.090	7 (6.67)	1 (6.25)	1 (4.54)	0.933
Diarrhea	15 (17.05)	2 (18.18)	2 (14.29)	0.960	8 (7.62)	1 (6.25)	2 (9.10)	0.947
Hemobilia	5 (5.68)	2 (18.18)	1 (7.14)	0.313	3 (2.86)	0	0	0.574
Delayed bleeding	3 (3.41)	0	0	0.645	2 (1.90)	0	1 (4.54)	0.605
Bile leakage	4 (4.55)	0	1 (7.14)	0.685	3 (2.86)	0	0	0.574
Jaundice	4 (4.55)	2 (18.18)	1 (7.14)	0.207	4 (3.81)	1 (6.25)	0	0.552
Wound infection	4 (4.55)	1 (9.09)	0	0.544	4 (3.81)	0	0	0.475
Sinus perforation	1 (1.14)	0	0	0.861	1 (0.95)	0	1 (4.54)	0.376

Abbreviations: CL=combined lithotripsy; EHL=electrohydraulic lithotripsy; IHD= intrahepatic duct; CBD=common bile duct.* $P<0.05$, † $P<0.01$.

Supplementary table S2. Complications between two groups in different impacted positions.

Variable	IHD			CBD			Basket		
	EHL (n=88)	CL (n=105)	***P***	EHL (n=11)	CL (n=16)	***P***	EHL (n=14)	CL (n=22)	***P***
**Intraoperation, No. (%)**	18 (20.45)	11 (10.48)	0.053	2 (18.18)	1 (6.25)	0.549	8 (57.14)	2 (9.09)	0.005†
Hemobilia	10 (11.36)	5 (4.76)	0.072	2 (18.18)	1 (6.25)	0.549	2 (14.29)	1 (4.54)	0.547
Cholangitis	11 (12.50)	7 (6.67)	0.165	0	0		6 (42.86)	2 (9.10)	0.036*
**Postoperation, No. (%)**	17 (19.31)	9 (8.57)	0.029*	4 (36.36)	2 (12.5)	0.187	6 (42.86)	3 (13.64)	0.111
Cholangitis	12 (13.63)	7 (6.67)	0.106	1 (9.09)	1 (6.25)	1.000	5 (35.71)	1 (4.54)	0.024*
Diarrhea	15 (17.05)	8 (7.62)	0.044*	2 (18.18)	1 (6.25)	0.549	2 (14.29)	2 (9.10)	0.634
Hemobilia	5 (5.68)	3 (2.86)	0.327	2 (18.18)	0	0.157	1 (7.14)	0	0.389
Delayed bleeding	3 (3.41)	2 (1.90)	0.841	0	0		0	1 (4.54)	1.000
Bile leakage	4 (4.55)	3 (2.86)	0.812	0	0		1 (7.14)	0	0.389
Jaundice	4 (4.55)	4 (3.81)	1.000	2 (18.18)	1 (6.25)	0.549	1 (7.14)	0	0.389
Wound infection	4 (4.55)	4 (3.81)	1.000	1 (9.09)	0	0.407	0	0	
Sinus perforation	1 (1.14)	1 (0.95)	1.000	0	0		0	1 (4.54)	1.000

Abbreviations: CL=combined lithotripsy; EHL=electrohydraulic lithotripsy; IHD= intrahepatic duct; CBD=common bile duct. * $P<0.05$, † $P<0.01$
